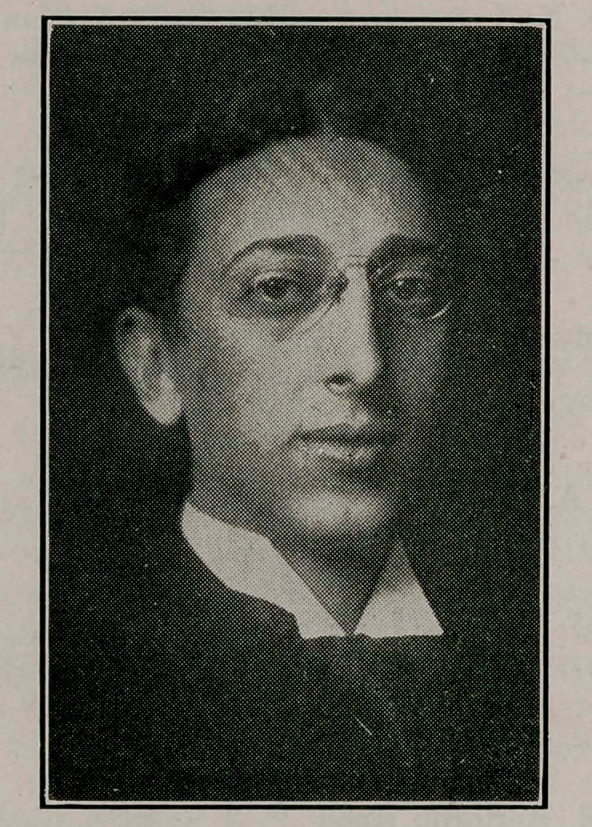# Frederick Carl Busch*An address delivered at the memorial hour of the 39th Annual Meeting of the Alumni Association of the Medical Department of the University of Buffalo, June 4, 1914.

**Published:** 1914-12

**Authors:** A. T. Kerr

**Affiliations:** Ithaca, N. Y.


### Frederick Carl Busch.*

DR. A. T. KERR, Ithaca, N. Y.

It is my privilege to have been selected to present to you today a tribute to my boyhood chum and life long friend and colleague, Dr. Busch, who for twelve years was Professor of Physiology in the Medical College here and in whose memory we are now assembled. It is most fitting that our memorial meeting should be in this building where he labored so long and so unselfishly and strove so earnestly to extend the bounds of knowledge and to elevate the standards of medical education. All realize that science and the medical profession have suffered a great loss in the passing, in the prime of his manhood and at the zenith of his powers, of an earnest and capable worker, but those of us who knew him personally as a physician, as a teacher, as an investigator, as a collaborator, as a colleague, as a friend, or as a man, appreciate even more deeply how irreparable this loss is.

Frederick Carl Busch was born in Buffalo, December 12, 1873, the oldest son of Frederick Busch and Kathlayne Layer (Busch). His father was born in Germany but in early life moved to Buffalo. His mother although born in this country was also of German parentage.

*An address delivered at the memorial hour of the 39th Annual Meeting of the Alumni Association of the Medical Department of the University of Buffalo, June 4, 1914.

Dr. Busch obtained his early education in the public schools of Buffalo graduating from Grammar School No. 16 in 1888 and from the Central High School in 1891. He was a good student and learned easily. The high school work in Chemistry and Physiology particularly aroused his interest and he fitted up in his own home a private laboratory in which to extend the experimental work beyond what was possible during school hours. The investigating and analytical qualities of his mind were early developed. I remember particularly at this period assisting him in some operations upon pigeons to relieve them of certain abnormal growths upon the head.

In the fall of 1891, to prepare himself more thoroughly for a medical career, he entered Cornell University in the science course, specializing, in the first year in Chemistry and in the later years of the course in the Biological Sciences, Zoology, Bacteriology, Histology and Embryology. While in college he was interested in athletics and was a competitor for the freshman crew and in the half mile run. Owing, however, to his rapid bodily development at this time, he did not prove a great success as an athlete and later in his course his energies were more centered in his scholastic work.

His lovable disposition made him popular with both teachers and students and he was a member of a number of student organizations including the Buffalo Club, the Cornell Medical Society, and the Beta Theta Pi Fraternity.

To supplement his university work he spent the summer vacation of 1903 at the Marine Biological Laboratory at Woods Hole in the study of marine invertebrates.

Because of additional work during his college course, he was able to finish all the requirements for the bachelor's degree in three and one third years and was therefore permitted by the faculty to be absent from the University during his senior year until April. Dr. Busch and I collaborated on our thesis which was entitled "The Muscles of the Thorax and Brachium of an Orang." In June, 1895, he graduated from Cornell University with the degree of Bachelor of Science.

In the summer of 1894, Dr. Busch started his medical studies in the Dispensary of the University of Buffalo where he worked mainly in the drug department. With the opening of the college year that fall, while on leave of absence from Cornell, he began his course in the Medical Department of the University of Buffalo under the preceptorship of Dr. Roswell Park. He served as student prosector in Anatomy for two years and was also appointed an assistant in the laboratories of Histology and Pathology. During the first year I worked with him upon an investigation of methods of determining the percentage of haemoglobin in the blood. The results of this work were presented before the American Microscopical Society and published in their proceedings, and in December of the same year in the Medical News there appeared another article on the same subject.

His connection with the department of Pathology continued throughout his medical course, and in addition to the regular medical studies he found the necessary time for teaching, for preparing specimens for class study in Pathology, for attending many autopsies and working up the post mortem material, and for conducting some research, in this way laying a solid foundation for his medical work.

In 1896 he published in collaboration with W. C. Keyes in the Buffalo Medical Journal a report of an interesting case of multiple echinococcus cysts.

At the time of graduation from the Medical College his thesis on "The Cultivation of the Gonococcus" won first prize in the Medical News competition open to all graduates of that year in the medical colleges of the United States.

Dr. Busch was a popular member of his class and in the senior year an officer. He was a member and president of the I. C. I. Society, now a chapter of the Nu Sigma Nu Fraternity.

After graduating in May, 1897, from the Medical Department of the University of Buffalo, he immediately began his service as resident physician in the Buffalo General Hospital, having won the appointment in competitive examination.

On June 22, 1898, after finishing his hospital work, he married Edith M. Fletcher of Middleport, N. Y.

He spent the summer of 1898 in the Marine Biological Laboratory at Woods Hole studying and experimenting in embryology. During the college year 1898-1899 he served as an assistant in the New York State Pathological Laboratory, now the State Institute for the Study of Malignant Diseases, and also as instructor in Physiology in the Medical Department of the University of Buffalo.

Early in 1899 having determined to devote his life to scientific work, Dr. Busch obtained a leave of absence from the University for the year and went abroad to continue his studies and fit himself for teaching and research in Physiology. How much determination it took to make this decision, how much of a sacrifice it meant to him, and how much faith in the future it required will be realized when it is known that he had no financial resources but was forced to borrow the money for the trip. The year abroad was spent mainly at the Physiologische Institut of the University of Berne, working with Professor Hugo Kronecker. While at Berne, four pieces of research were completed on the resonance of nerve and muscle; "Fibrillation and Pulsation of the Dog's Heart;" "Propogation of Impulses in the Rabbit's Heart;" "Die Eigenshaften und die Enstehung der Lymphe."

Upon returning to Buffalo in the fall of 1899, Dr. Busch was appointed Professor of Physiology in the Medical Department of the University of Buffalo and was given complete charge and responsibility for the physiological work in the Medical School. Up to this time the work in physiology for medical students here as in most other medical schools in this country, had consisted almost entirely of lectures and recitations supplemented by experimental demonstrations by the teacher before the class. But medical education was changing and it was now seen to be important that the students should not only be told and shown but that they should think and do things for themselves. This meant the establishment of modern laboratories for student teaching. The endless details involved in organizing and equipping laboratories for classes and for research can only be realized by those who have undertaken similar work. How well Dr. Busch accomplished this task, at the same time carrying on his lectures, class teaching, and research. you all know.

From this time until his resignation two years ago to undertake other research work he devoted himself without stint to his work as a teacher and investigator, maintaining his courses at all times on a high plane of excellence and keeping well in the fore of the rapid advance in medical teaching.

But in addition to his work as a teacher and investigator, in order to patch out his meagre salary it was necessary that he should also practice medicine, and although he kept some office hours and saw a few patients this was but an incident in his daily life which was devoted mainly to his college. His deep sympathies combined with his thorough training and keen mind gave him an unusual insight into diseased conditions and his personality attracted patients to him. Had he chosen to devote himself exclusively to medicine, he would have been a most successful practitioner with a large following.

The next twelve years were busy but happy ones, for although teaching and practice took up much of his attention, there was still some time for research. That Dr. Busch was a diligent worker will be seen from the number and importance of the papers published by him during this period.

Among these may be mentioned his articles on the haemoglobin of the blood, upon the leucocytes of the dog's and cat's blood; upon the lymphomatous tumors of the dog's spleen; upon pancreatitis; upon the effect of olfactory stimuli on sweating; the action of diuretics upon excised kidneys; and his well known work on the suprarenal glands and their transplantation with preservation of function; his work upon Addison's disease; and finally his experimental work on the arrest of hemorrhage by means of blood transfusion and serum injections resulting ultimately in the preparation with Dr. Slewes of a dried precipitated blood serum for the arrest of hemorrhage now known as "coagulose." He is at present best known for his work on the suprarenals, but the importance of his work on coagulation of the blood is attaining more and more recognition.

His investigations were painstaking and thorough with far reaching results so that he had attained a no mean reputation among the physiologists both in this country and abroad. His investigations were, as a rule, undertaken in collaboration with other workers to whom his enthusiasm and originality were a constant inspiration.

In November, 1910, he again joined the staff of the State Institution for the Study of Malignant Diseases although still continuing his work as a teacher of Physiology in the University. In connection with his work at the Institute the summer of 1911 was spent at the Craig Brook Fish Hatchery in Maine working upon carcinoma of the thyreoid in Salmonoid fishes.

He had been interested in the State Institute for Malignant Diseases since its foundation and had kept in close touch with the investigations there underway. The broader scope of the work which the Institute was now undertaking, including the diseases of internal secretion, upon the physiology of which he had long been working, and the addition to the laboratory of a hospital for the treatment of these and other malignant diseases, caused him to see the unusual opportunities for research in his chosen field thus opened to him. He realized, however, that in order to accomplish the most for the almost hopeless sufferers from these maladies he must devote himself entirely to the study and treatment of these diseases, so in 1912 he resigned his professorship in the University of Buffalo in order to give his whole time to this new work.

To prepare himself to take charge of the clinical work in the hospital of the Institute, he went to Europe in the fall of 1912. While abroad he worked mostly in the hospitals and laboratories in Berlin and also acquainted himself with the action of and method of handling radio-active substances.

Upon his return from abroad, although then in the grip of the malignant disease which ultimately caused his death, he continued his work and arranged and planned the record system for the hospital. He had in prospect some interesting researches in metabolism with promise of important results. Active symptoms of his disease long ignored now became insistent and in November he went to Baltimore to place himself in the hand of specialists there. Operation revealed an extensive cancer of the bladder which was treated by cautery and a massive dose of radium. He was slightly improved and late in December returned to Buffalo to the hospital of the

New York State Institute for Malignant Diseases in which it was originally planned that he was to have charge of the clinical work. He died on January 3, 1914, of a terminal pneumonia. Surviving him are his wife and seven year old son, Addison Fletcher Busch, his mother, one brother, George A. Busch, and a sister, Mrs. James Traunce.

Those of you who knew him as a teacher will remember the clearness of his exposition and the charm of his presentation. He possessed the rare faculty of conveying complicated scientific truths in simple language that made it interesting to all who heard him. The effectiveness of his teaching was heightened by the unusual point of view, by the quaint stories, often humorous, by which he impressed a point, and the earnestness of his bearing was relieved, especially at these times, by that inimitable genial smile. The magnetism of his manner and the quiet but contagious enthusiasm coupled with an unconventional, modest, and affable bearing endeared him to all. It was not alone as a lecturer that he excelled but in the laboratory where he seized the opportunity to train his students to observe closely and to reason clearly but above all to become independent thinkers and workers. He was most approachable and those who came to him for help know how painstaking and unwearying were his efforts to aid. He liked to teach and was an inspiring teacher with a strong and enduring influence upon his students. It was only because he felt that his training and experience had especially fitted him to undertake a greater work in the institute for Malignant Diseases, with greater opportunities for service, that he relinquished his teaching position in the University here.

He was naturally a fluent speaker and writer of English. He wrote and spoke also French and German, the latter almost like a native. Probably his facility in language was influenced, not only by inheritance but also by his early training, for as a very young child he learned to speak the German language although he did not keep up the practice in his youth.

Dr. Busch was pre-eminently an investigator with fine natural endowments and an aptitude for knowledge. He was indefatigable in devotion to his work, a clear, deep thinker with a broad view, a conscientious critic, forceful and discriminating with a philosophical balance. Above all discouragements he was true to his ideas, to his ideals, to truth, and to his task, and he has gathered a rich harvest of discovery. It was a pleasure to see him accomplish results with so little fuss. Although his interests were mainly in pure science, he was trained as a physician and the application of his science to the cure of disease, and the alleviation of suffering was always uppermost in his mind.

Dr. Busch was widely popular with broad interests in many fields. He was a member of many societies and organizations, civic as well as scientific, among these latter were the American Association for the Advancement of Science; the American Medical Association; the Medical Society of the State of New York; the Medical Society of Erie County; the Roswell Park Medical Club; the Buffalo Medical Club; the Buffalo Academy of Medicine; the Buffalo Society of Natural Science; the Buffalo Historical Society; the American Society for the Encouragement of Research; and the American Physiological Society. The social organizations with which he was closely associated include the University Club, Westminster Club; Guido Chorus, and he maintained throughout his life an active interest in his college societies Nu Sigma Nu, Beta Theta Pi, and Sigma Xi.

It was not until about 1899 that Dr. Busch, urged by his friends, began training his voice, which has delighted so many Buffalonians and has given so much joy to his friends. Later he became a member of the quartette of the Westminster church; and as one of the soloists of the Guido Chorus, was widely known. He had a splendid baritone voice, of fine quality and rare sweetness, but it appealed especially because of his sympathetic interpretations. He was a musician in the purest sense of the word and one felt that it was never an effort for him to sing or play but that he was getting out of it as much pleasure as he was giving.

The best human characteristics were exemplified in Dr. Busch's fine qualities. Gentle, modest, unselfish, generous, frank, loyal, his was one of the purest characters with a charming simplicity of manner and an absence of worldliness seldom seen. These fine qualities were coupled with his natural endowments, scholarship, culture, and refinement; love of poetry, art, and music, to make a perfect gentleman. Few realized his strong religious faith and rich Christian life. Agony and suffering he bore with fortitude and control, always considerate of his friends before himself. A chosen spirit, a rare man, admired and beloved by all. To those of us who were privileged to have known him, his life so short, so full, will always stand out as a shining example of what we should most like to be.

1895 Comparison of the Fleishl, the Gower, and the Specific Gravity Methods of Determining the Hemoglobin of the Blood for Clinical Purposes. Proc. Am. Mic. Soc., vol. XVII.

1895 The Relations between the Specific Gravity of the Blood and its Hemoglobin Percentage. Medical News, Dec. 21, 1895.

1895 A Case of Multiple Echinococcus Cysts. Buffalo Medical Journal, August, 1896.
1896 On the Cultivation of the Gonococcus. Medical News, December 1896. (Prize essay—1st prize.)
1899 On the Resonance of Nerve and Muscle. British Assoc. Report, 1899.
1899 Concerning Fibrilation and Pulsation of the Dog's Heart. British Assoc. Report, 1899.
1899 The Propagation of Impulses in the Rabbit's Heart. British Assoc. Report, 1899. With Prof. Hugo Kronecker.
1900 Untersuchungen ueber die Eigenshaften und die Entstehung der Lymphe. With Dr. Leon Asher. Zeitschr. f. Biologie, Bd. XL.
1900 The Relation of the Specific Gravity of the Blood to its Percentage of Hemoglobin. With Dr. Kerr and Dr. Filsinger. Buffalo Medical Journal, October, 1900. Also in Report I, Pathol. Lab. University of Buffalo.
1902 The Lymphomatous Tumors of the Dog's Spleen. With Dr. Herbert U. Williams. The Journal of Medical Research, May, 1902, vol. VII, No. 4, pp. 408-410.
1902 Dog's Blood—Differential Counts of the Leucocytes. With Dr. Van Bergen. The Journal of Medical Research, November, 1902, vol. VIII, No. 2.
1903 Cat's Blood—Differential Counts of the Leucocytes. With Dr. Van Bergen. The Journal of Medical Research, vol. X, No. 2 (N. S. Vol. V, No. 2), pp. 250-254.
1904 Editor of the Fifth American Revision of Kirke's Handbook of Physiology. Wm. Wood and Company, New York.
1905 Laboratory Manual of Physiology. IX, 206 pp. ill. Wm. Wood and Company.
1906 Suprarenal Transplantation with Preservation of Function. With Dr. Charles Van Bergen. The Am. Jour. of Physiology, vol. XV, April 2, 1906, No. 5.
1907 The Etiology and Pathogenesis of Acute Pancreatitis. With Dr. Herbert U. Williams. The Journal of Medical Research. Vol. XVIII, No. 1, October, 1907.
1908 Further Results in Suprarenal Transplantation. With Dr. Theodore M. Leonard, and Dr. Thew Wright. The Journal of the American Medical Association, August 22, 1908, Vol. LI, pp. 640-642.
1909 Localized Facial Sweating, Following Certain Olfactory Stimuli. With Dr. Grover W. Wende. The Journal of the Amer. Med. Assoc., July 17, 1909, Vol. LIII, pp. 207-208.
1910 Three Cases of Addison's Disease, one with Adrenal

Transplantation. With Dr. Thew Wright. Archives of Internal Medicine, January, 1910, Vol 5, pp. 30-36.

1913 Treatment of Hemorrhage by Means of Precipitated Blood Sera. With Dr. S. H. A. Clewes.

1914 Carcinoma of the Thyreoid in the Solmonoid Fishes. Collaborator with Harvey R. Gaylord, Millard C. Marsh, Burton T. Simpson, 162 pp. 127 figures (9 in colors) from Bulletin of the Bureau of Fisheries, vol. 32, 1912. Document No. 790, issued April 22, 1914, Government Printing Office, Washington, D. C.

## Figures and Tables

**Figure f1:**